# Alkaptonuria: From Molecular Insights to a Dedicated Digital Platform

**DOI:** 10.3390/cells13121072

**Published:** 2024-06-20

**Authors:** Maria Serena Milella, Michela Geminiani, Alfonso Trezza, Anna Visibelli, Daniela Braconi, Annalisa Santucci

**Affiliations:** 1 ONE-HEALTH Lab, Department of Biotechnology, Chemistry and Pharmacy, University of Siena, 53100 Siena, Italy; serena.milella@gmail.com (M.S.M.); alfonso.trezza2@unisi.it (A.T.); anna.visibelli2@unisi.it (A.V.); daniela.braconi@unisi.it (D.B.); annalisa.santucci@unisi.it (A.S.); 2 SienabioACTIVE-SbA, Department of Biotechnology, Chemistry and Pharmacy, University of Siena, 53100 Siena, Italy; 3 ARTES 4.0, Department of Biotechnology, Chemistry and Pharmacy, University of Siena, 53100 Siena, Italy

**Keywords:** amyloid, artificial intelligence, biomarkers, cartilage, inflammation, machine learning, ochronosis, osteoarthritis, oxidative stress, rare disease

## Abstract

Alkaptonuria (AKU) is a genetic disorder that affects connective tissues of several body compartments causing cartilage degeneration, tendon calcification, heart problems, and an invalidating, early-onset form of osteoarthritis. The molecular mechanisms underlying AKU involve homogentisic acid (HGA) accumulation in cells and tissues. HGA is highly reactive, able to modify several macromolecules, and activates different pathways, mostly involved in the onset and propagation of oxidative stress and inflammation, with consequences spreading from the microscopic to the macroscopic level leading to irreversible damage. Gaining a deeper understanding of AKU molecular mechanisms may provide novel possible therapeutical approaches to counteract disease progression. In this review, we first describe inflammation and oxidative stress in AKU and discuss similarities with other more common disorders. Then, we focus on HGA reactivity and AKU molecular mechanisms. We finally describe a multi-purpose digital platform, named ApreciseKUre, created to facilitate data collection, integration, and analysis of AKU-related data.

## 1. Oxidative Stress and Chronic Inflammation in Human Diseases

Reactive oxygen species (ROS) are physiological products of mitochondrial metabolism that, when finely tuned, participate in many cellular and developmental processes, including intracellular signalling and immune response [1,2]. Excess ROS can be generated in pathological contexts as a defence mechanism against environmental stimuli [2,3,4]. The disbalance between production of ROS and their detoxification/removal by antioxidants generates a condition known as “oxidative stress” [5], potentially detrimental for biological macromolecules [6].

Inflammation is a complex defence mechanism activated in response to pathogens, infections, or dangerous endogenous signals characterized by the accumulation of monocyte-derived macrophages and lymphocytes, high circulating levels of cytokines and chemokines, and the proliferation of fibroblasts and small blood vessels [7,8]. Chronic, unregulated inflammation due to the body’s inability to remove the triggering stimulus is associated with several human diseases [9] and has potentially fatal consequences [10].

Current knowledge indicates an intimate, interdependent relationship between oxidative stress and inflammation that can create a vicious cycle [7,11,12]. This interplay is exemplified by NF-κB proteins, a family of transcription factors of central importance in inflammation whose activity can also influence ROS levels [3], as well as by the NLRP3 inflammasome, a molecular complex for which ROS can serve either as triggering or effector molecules [13]. Several human pathological conditions present with oxidative stress and inflammation [14,15], such as neurodegenerative [16], autoimmune [17], rheumatic [18], and cardiovascular diseases [19], as well as cancer [20,21].

### Oxidative Stress, Chronic Inflammation, and Secondary (AA) Amyloidosis in Rheumatic Diseases

Oxidative stress and inflammation are common players in rheumatic diseases such as osteoarthritis (OA) [9,22,23] and rheumatoid arthritis (RA) [24,25,26]. OA is the most prevalent joint degenerative disease observed in the aging population, characterized by irreversible structural and functional changes in joints leading to severe pain, deformity, disability, and reduced quality of life (QoL) and life expectancy [9]. Increased levels of inflammatory mediators as well as accumulation of ROS characterize OA joint tissues and lead to abnormalities [22]. At the cellular level, several lines of evidence suggest a critical and complex role of NRF2 in chondrocytes and osteoblasts/osteoclasts metabolism [22,23]. RA is a rheumatic autoimmune disease with a complex aetiology, as it involves the release of pro-inflammatory cytokines that infiltrate the synovial joint in a self-amplifying loop, leading to production of autoantibodies, cartilage destruction, bone resorption and protein modifications, and overall enhancement of chronic inflammation [26]. In parallel, oxidative stress is elicited through mitochondrial dysfunction [27] and NRF2 involvement [28] contributing to pathological mechanisms.

A rare complication of rheumatic diseases including RA, juvenile idiopathic arthritis, and ankylosing spondylitis is secondary (AA or reactive) amyloidosis [29,30]. It involves the deposition of amyloid fibrils, generated from serum amyloid A (SAA) proteins, in several tissues such as the kidney, gastrointestinal tract, and heart [29]. SAA is an acute-phase inflammatory apolipoprotein, released mainly by the liver following TNF-α and IL-1β induction, whose systemic levels are significantly increased during chronic inflammation. SAA is also produced extra-hepatically, and local production in synovial tissues has been demonstrated, suggesting a relationship with angiogenesis, leukocyte infiltration, and synovial invasion in RA [31]. Once internalised by cells and accumulated in vesicles, SAA spontaneously undergoes self-assembly into amyloid aggregates having a β-lamina shape [32], affecting cellular survival. Release into the extracellular space allows the amyloid deposits to grow, ultimately leading to amyloid fibrils [33]. The amyloid progression is slow and characterized by organ failure, which ultimately affect patients’ life expectancy [34]. Amyloid fibrils can also contain serum amyloid P (SAP), a pentameric protein binding amyloid precursors acting as a stabilizer by preventing proteolytic cleavage [35]. SAA can act as a strong pro-inflammatory mediator, inducing the secretion of cathepsin B from human macrophages and the activation of the NLRP3 inflammasome [36]. Additionally, SAA can promote expression of adhesion molecules, angiogenesis, and matrix degradation via NF-kB mediated mechanisms [31]. SAA and its signalling pathways represent, therefore, novel potential therapeutic targets when the inflammatory stimulus cannot be removed.

## 2. Molecular Insights into Alkaptonuria

### 2.1. Alkaptonuria

Alkaptonuria (AKU) is a rare genetic disease caused by the progressive accumulation of homogentisic acid (HGA) in the body. HGA is an intermediate in the catabolic pathway of tyrosine that should be broken down into 4-maleyl acetoacetate by homogentisate 1,2-dioxygenase (HGD), an enzyme mainly expressed in the liver and kidneys [37]. To date, more than 200 different disease-associated *HGD* variants, mostly missense substitutions, have been described in more than 700 AKU patients [37]; these mutations lead to malfunctioning HGD enzymes with different residual activities causing accumulation of HGA in body fluids and tissues. However, genotype–phenotype studies in AKU patients have shown, so far, no clear correlations [37]. Excess HGA undergoes spontaneous auto-oxidation and polymerization reactions in vitro [38], producing a black pigment known as ochronotic pigment. In vivo, the HGA-derived ochronotic phenomenon is observed in affected patients’ body fluids, as exemplified by the darkening of alkaptonuric urine exposed to oxygen or alkali [39], as well as in patients’ collagenous tissues. Circulating HGA is largely excreted through urine [40], though there is a non-excreted portion that accumulates in joints and organs, leading to the formation of bluish-black patches characterized by the presence of the HGA-derived black pigment (ochronosis). The homogentisic aciduria occurs from birth and causes blackening of urine, which is the first sign of AKU during this first asymptomatic stage of the disease. It is during the third or fourth decade of life that the most debilitating arthritis-like symptoms appear. Diagnosis of AKU is reached based mainly on these two features of the clinical history; however, the non-specific nature of symptoms along with a general lack of knowledge among paediatricians and physicians can significantly delay the time of diagnosis.

**Figure 1:**
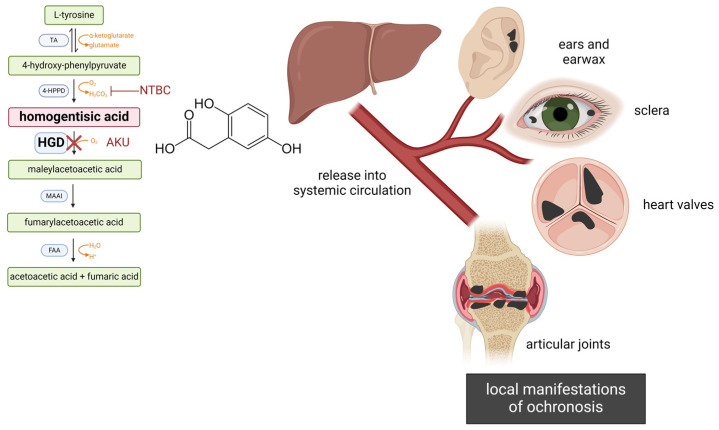
A malfunctioning HGD enzyme (indicated with a red cross) in the tyrosine metabolic pathway (**left**) causes accumulation of HGA. The drug nitisinone (NTBC) inhibits the upstream 4-HPPD enzyme, thereby blocking HGA production. In untreated AKU, excess HGA is released (mostly from the liver) into systemic circulation and delivered to target tissues, leading to ochronosis (i.e., deposition of a black, melanin-like pigment). Ochronosis affects several body compartments (**right**), with serious consequences especially for load-bearing articular joints, heart valves, and aortic vessels. Enzyme abbreviations: TA, tyrosine aminotransferase; 4-HPPD, 4-hydroxyphenylpyruvate dioxygenase; HGD, homogentisate 1,2-dioxygenase; MAAI, maleylacetoacetate isomerase; FAA, fumaryl acetoacetase. Created with BioRender.com (https://www.biorender.com/, accessed on 17 June 2024).

**Figure 2:**
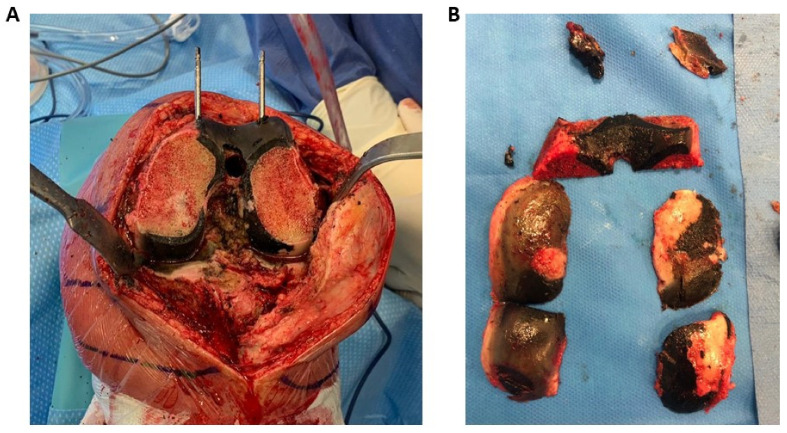
The knee of an AKU patient during arthroplasty (**A**). Ochronosis involves the whole joint, including ligaments and tendons that appear black; the cartilage is extremely thin, brittle, and fragile (**B**).

The deposition of the ochronotic pigment in the body is not uniform: most tissues are resistant to the pigmentation, and ochronosis mostly affects connective tissues, as suggested by preliminary studies on animals [41]. Articular cartilage, joints, spine, tendons, and ligaments are strongly pigmented in AKU. Pigmentation is also visible in the eye sclera, ear cartilage and wax, aortic roots and valves, glands, skin, and sweat (Figure 1). Pigmented tissues are significantly damaged, with severe/complete loss of function. Due to the presence of the ochronotic pigment, AKU cartilage is thin, stiff, and brittle, susceptible to fracture and splinter. Several degenerative changes, including joint-space narrowing, cartilage irregularities, subchondral sclerosis, and peripheral osteophytes can be detected during radiological inspection [42]. Similarly, ochronosis makes tendons and ligaments more prone to ruptures [43]. At the microscopical level, AKU cartilage is characterized by the formation of dystrophic lacunae and by the substitution of collagen type II with type III, which assumes a disorganized arrangement [44]. The ochronotic arthropathy that ensues is responsible for pain and loss of function of large joints [45], and, due to irreversible destruction, patients commonly need total replacement surgery of the knees, hips, and shoulders (Figure 2). The spine is impaired as well with degeneration and calcification of the intervertebral discs, narrowing of intervertebral spaces, and formation of bony bridges between vertebrae [46]. AKU patients have difficulty in walking and in movements, with a reduction in the range of possible motions [47]. AKU is also associated with scoliosis, kyphosis, and spondylosis, which causes a decrement in patient height [48].

The cardiovascular system can be impacted as well by ochronosis. Cardiac valves, vessels, endocardium, and intima can become strongly pigmented, and the aortic valves can become thickened and calcified [49]. Over time, valves lose their functions and must be replaced surgically, an early marker for reduced cardiovascular health being an impaired aortic distensibility [50]. Data obtained in a AKU cohort revealed that all patients older than 65 years had aortic stenosis, while in the group of individuals over 50 years, 10% had aortic sclerosis and 40% had aortic stenosis [51].

AKU patients can also be subjected to the development of renal and prostate stones [8]. In case of renal failure, renal transplantation can not only restore HGA excretion with urine, but also provide functioning HGD necessary for the correct metabolism of HGA [52].

### 2.2. HGA, Ochronosis, and Oxidative Stress

The ochronotic pigment is also referred to as melanin-like pigment because both derive from tyrosine and have similar absorbance spectra, with a peak in the UV-visible range [53,54]. It settles preferentially in connective tissues, especially cartilage, in correspondence with collagen fibres. It seems that the loss of protective molecules such as proteoglycans and glycosaminoglycans, which is a natural ageing process but can be even more relevant in AKU, can unmask binding sites for HGA or its byproducts in collagen fibres [53]. Hence, pigment subunits accumulate, triggering the rapid formation and deposition of ochronotic deposits. In AKU, the cytoskeleton is severely compromised too, showing an aberrant expression of the three most important cytoskeletal markers (β-actin, β-tubulin, and vimentin) probably leading to a progressive loss in cartilage architecture due to the disorganisation of the extracellular matrix (ECM) [55]. Higher amounts of type III collagen in place of type II collagen can be detected in AKU tissues, indicating matrix damage similar to OA [56]. It has been suggested that type III collagen may provide cohesion to a type II fibril network significantly weakened by the HGA-induced stress [44].

The composition of the ochronotic pigment remains quite obscure. To fill this gap, several efforts were made over the last decades to clarify the chemical reactivity of HGA. One of the previously most accredited hypotheses for the fate of HGA was its oxidation into a quinone derivative prone to generation of ROS and oxidative stress [57], reaction with biological amines, and polymerization into a pyomelanin-like structure building up in connective tissues [58]. The electrochemical behaviour of HGA [59,60,61,62], along with the structure and properties of the HGA-derived pyomelanin [63,64] have been reported. In this framework, it was found that the in vitro polymerization of HGA under pathologically relevant conditions could generate different melanin-like polymers [65,66]. More recent work, corroborated by the analysis of AKU biofluids, seemed to rule out the existence of an HGA-derived quinone and rather suggested deprotonation and loss of carbon dioxide from HGA along with the generation of free radical intermediates and propagation of oxidative stress [38,67].

Despite being informative, in vitro chemical studies on HGA suffer the main limitation of the lack of a complex biological context fully recapitulating the pathological setting. To overcome this limit, several in vitro and ex vivo models of AKU were investigated, showing that the presence of HGA in cells or tissues is paralleled not only by increased oxidative stress, but also persistent low-grade inflammation and impaired protein aggregation leading to secondary amyloidosis [68,69,70,71,72,73,74,75,76,77,78,79,80,81,82,83,84]. Several oxidative protein post-translational modifications (PTMs) were associated with the presence of HGA [85], including protein carbonylation [70,79] and the formation of high molecular weight carbonylated aggregates [69], thiol-oxidation [78,86], lipid peroxidation [55], and 4-HNE protein modification [87], as well as glycation and nitrate addiction [80]. Some of these PTMs are irreversible and likely to cause significant structural and functional alterations on affected proteins; alterations of the protein folding might lead to an increase in the hydrophobic exposed surface making proteins more prone to aggregation.

Oxidative stress in AKU was ascertained in vitro in HGA-treated human serum where lipid peroxidation, decreased glutathione peroxidase activity, massive depletion of thiol groups, and the carbonylation of serum proteins were detected [78,79,88]. A further analysis on serum proteins that could be oxidatively modified by HGA suggested alterations of metal homeostasis and protein aggregation [78,79]. Interestingly, differences were highlighted in HGA-induced carbonylation patterns against important anti-oxidant serum proteins such as ceruloplasmin and transferrin, whose consequences deserve investigation [75]. A comparative proteomic study on serum and plasma samples from alkaptonuric individuals showed clear alterations in the abundance of proteins involved in stress defence mechanisms, damage repair, and pathways involved in programmed death, with the expression of several apolipoproteins, glycoproteins, complement factors, and protease inhibitors compared to healthy individuals [77].

In HGA-treated human chondrocytes, proteomic studies indicated that HGA could affect the abundance of proteins with a role in protein folding, cell organization, cell defence, and stress response [79]. This result was corroborated with the proteomic analysis of chondrocytes from alkaptonuric donors, showing an aberrant abundance of proteins involved in protein fate, cell structure and organization, cell rescue, defence, and stress response [68]. These findings suggest that cells react to HGA-induced oxidation by activating pathways related to stress responses; however this activation might be insufficient to restore a normal condition, allowing propagation of oxidative damage and cell apoptosis [89].

Another relevant HGA-induced alteration found in AKU is ciliopathy [90,91], since shorter and structurally altered primary cilia were found in alkaptonuric and HGA-treated cells compared to their healthy counterparts [90,91], in good agreement with the previously discussed HGA-induced cytoskeletal aberrations [55]. It is known that primary cilia are key mechano-sensory organelles in chondrocytes [92] whose integrity is needed for cartilage development and homeostasis [93], mechanosignalling [94], inflammatory NF-kB signalling [95], and Hedgehog (Hh) signalling [91,92]. Hh signalling regulates many developmental and physiological processes and is activated during tissue damage repair [96,97]. In OA chondrocytes, Hh signalling disruption might promote cartilage degradation through ECM degrading enzymes [97] and is correlated with OA severity [98,99,100,101]. Therefore, HGA might be responsible in AKU for the defective regulation of ciliary trafficking and the inability to activate Hh signalling upon exogenous ligand binding, which can contribute to the development of ochronotic arthropathy [91]. Hh also regulates autophagy [102], a fundamental process for the degradation of damaged organelles and proteins. It was therefore interesting to note that disrupted autophagy was found in AKU [89], possibly promoting degeneration of cartilage similar to OA, especially in load-bearing joints [103] and cell apoptosis [89]. In summary, excess HGA in AKU can create a vicious crosstalk for pigment formation, tissue disruption, and disease progression mediated by oxidative stress.

### 2.3. HGA, Inflammation, and Amyloidosis

As previously discussed, oxidative stress is strictly intertwined with inflammation. Similarly to other diseases, ROS and their oxidant products might cause the activation of inflammatory signals in AKU as well. Given the chronic nature of the disease, the HGA-derived inflammatory stimulus is therefore likely to persist.

Previous research has shown that AKU chondrocytes are characterized by significantly higher levels of IL-1β, IL-6, IL-8, IL-10, and TNF-α compared to non-AKU cells [68,84], supporting the inflammatory nature of the disease. Proinflammatory cytokines are also more abundant in alkaptonuric plasma [82] and in an HGA-treated cell model [83] compared to control counterparts. Specifically, a positive correlation between serum levels of IL-6 (a key factor in the stimulation of immune and inflammatory response), age, and AKU severity was highlighted [104]. Excess HGA is a persistent stimulus in AKU, causing a chronic subclinical inflammation both at the local and systemic level, ultimately leading to the onset of ochronotic arthropathy. This condition is similar to what is observed in other rheumatic conditions, including OA. Therefore, the study of the molecular mechanisms underlying the activation of inflammatory signalling in AKU by means of disease models might offer novel clues for better disease management, possibly delaying the occurrence of symptoms and allowing improvement of patients’ QoL.

Reactive (secondary, AA) amyloidosis was recently identified in AKU as a consequence of increasing circulating serum amyloid A (SAA), an inflammatory protein. Indeed, in the majority of AKU patients, serum SAA was >10 mg/L [75,76,77]. SAA-related amyloid deposits were found in different tissues from several alkaptonuric donors, including cartilage, tendon, synovia, periumbilical fat, and salivary glands [71,81,83]. The cardiovascular system was affected too, and amyloid fibrils were detected in aortic valves [72,82]. Interestingly, amyloidosis was also found in tissues from young, even asymptomatic AKU subjects [44]. This suggests that over expression of SAA might occur in early AKU stages. In both human chondrocytes and articular cartilage, exogenous supplementation with HGA causes the formation and deposition of amyloid [37]. Furthermore, histological analyses showed that SAA colocalizes with cytoskeletal markers, probably leading to disorganization of the ECM [55]. Hence, with HGA being a chronic stimulus in AKU, the progressive accumulation of amyloid fibres in tissues can lead to tissue degeneration. Reflecting the multisystemic nature of AKU, amyloidosis can relentlessly target various organs, progressively causing dysfunction and failure and contributing to stiffness, swelling, and limitations in movement, with a progressive decline in a patient’s overall health and QoL. Interestingly, SAA plasma levels may represent a promising prognostic marker in AKU since they are correlated with disease severity [83].

**Figure 3:**
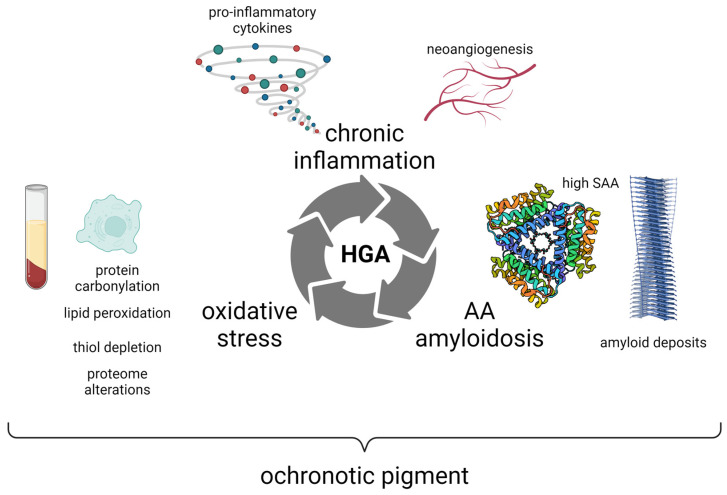
In AKU, oxidative stress, chronic inflammation, and secondary (AA) amyloidosis are strictly interconnected in a vicious cycle linked to the production and deposition of the ochronotic pigment derived from HGA. Created with BioRender.com (https://www.biorender.com/, accessed on 17 June 2024).

High vascularization and expression of endothelial markers were also observed in an ex vivo synovium model obtained from alkaptonuric patients, indicating neoangiogenesis [105]. This phenomenon is likely to contribute to an increased supply of oxygen, HGA, and inflammatory proteins such as serum amyloid A (SAA) [75,76] in proximity of ochronotic pigment patches, hence promoting oxidative stress, inflammation, aggregation, and further ochronotic pigment deposition [105] (Figure 3).

SAA amyloid deposits and ochronotic pigment patches co-localize in AKU tissues [83]. Current knowledge cannot clarify if the ochronotic pigment promotes the formation of amyloid aggregates or if, vice versa, the amyloid fibrils act as a scaffold facilitating pigment deposition. In the former case, it can be therefore speculated that amyloid might play a functional role, engulfing the pigment to shield the surrounding areas from its toxic oxidative effects. If, on the other hand, amyloid deposits are formed solely because of high circulating SAA, the deposition of the ochronotic pigment can follow simply due to physical/chemical interactions. In any case, the two processes are strictly intertwined and affect each other. For instance, it was found that HGA could act in vitro as an enhancer of SAA aggregation in a time- and dose-dependent manner [73]. The relationship between ochronotic pigment and amyloid fibrils is still under investigation, and it could provide novel clues for the understanding of the molecular mechanisms of AKU.

### 2.4. In Vivo Studies

Prompted by in vitro evidence linking HGA, oxidative stress, inflammation, and amyloidosis, as discussed in the previous paragraphs, research was undertaken to verify in vivo these findings. The opportunity to do so was provided by several clinical studies that allowed investigating the presence of several oxidative and inflammatory biomarkers in blood samples obtained from alkaptonuric individuals. Among such studies, it is important to mention two recent, relatively large randomized international clinical trials, i.e., SONIA1 and SONIA2 (Suitability Of Nitisinone In AKU), to assess the efficacy and safety of the drug nitisinone (NTBC) in AKU [106,107], as well as SOFIA (Subclinical Ochronosis Features In AKU), a longitudinal observational study to establish when ochronosis starts [108].

Serum total antioxidant capacity, protein carbonyls, and advanced oxidation protein products (AOPP) were found to be higher in AKU compared to control subjects in small cohorts of alkaptonuric subjects [77,104]. However, these findings were not confirmed in SONIA1 and SONIA2, where AOPP levels were almost exclusively in a non-pathological range [75,76]. Similarly, in SONIA1, serum thiols, S-thiolated proteins, and the Protein Thiolation Index (PTI) showed no statistically significant differences from control populations [76] despite previous contrasting evidence suggesting increased PTI values in a very-small group of alkaptonuric individuals [109]. Nevertheless, proteomic analyses confirmed the pro-oxidant action of HGA against human serum proteins in vivo [77].

In SONIA 1, inflammatory biomarkers such as CRP IL-1b, IL-6, and TNF-α showed no statistically significant differences in AKU compared to controls, whereas chitotriosidase activity (a marker of macrophage/monocyte activation) was significantly increased in nearly half the studied population [76]. SAA was found to be consistently elevated in AKU in SONIA1 and SONIA2 patients as well as in other smaller AKU populations [74,75,76,77,82,83,84]. Even if it was shown that both SAA and the PTI increased with age in AKU, with a trend similar to non-AKU controls [108], SAA and chitotriosidase activity showed a significant, positive correlation with AKU disease severity [75,76].

### 2.5. From Molecular Insights to Therapeutic Approaches

NTBC, 2-(2-nitro-4-trifluoromethylbenzoyl)-1,3-cyclohexanedione, is currently the only approved drug for treatment of AKU [106]. Other than that, only palliative therapy including drugs to control pain and inflammation, as well as arthroplasty, are available to alkaptonuric patients.

NTBC potently inhibits an upstream enzyme of HGD in the tyrosine catabolic pathway, i.e., 4-hydroxyphenylpyruvate dioxygenase (4-HPPD), thereby preventing formation and accumulation of HGA in biological fluids [106,107]. NTBC is associated with severe side effects due to increased tyrosinemia, including leukopenia, thrombocytopenia, and ocular complications [37]. Furthermore, research has shown that ochronosis may start well before other clinically relevant symptoms [108], implying that NTBC should actually be started ideally during childhood, which would pose unacceptable risks for a usually non-life threatening disease such as AKU [37]. In SONIA1, it was found that NTBC had no significant effects on serum CRP, IL-8, SAA, and AOPP after a 4-week treatment, whereas in SONIA2 NTBC, it was associated with a slight increase in circulating SAA and higher patient body mass index after 4-year treatment [75]. Therefore, despite being extremely effective in reducing HGA levels, NTBC still appears as an imperfect therapy.

Based on the knowledge of the molecular mechanisms of AKU, it seems reasonable to explore alternative therapeutic approaches that could potentially represent important co-treatments for AKU. For instance, methotrexate [83] and antioxidants [84] could inhibit the production of amyloid and the release of pro-inflammatory cytokines in a chondrocyte-based disease model, suggesting their potential use in AKU. Inhibitors of the Smo receptor could target Hh pathway activation and primary cilia structure, which are affected by HGA in AKU [90,91]. Computational in silico approaches exploring the formation and druggability of transient pockets in the HGD enzyme might help the identification of novel pharmacological molecular chaperones assisting a correct protein folding [110,111]. In addition, novel NTBC analogues with improved pharmacokinetics and pharmacodynamics could enable a better modulation of HPPD activity [112,113,114,115], while approaches based on tyrosine ammonia lyase [116] or inhibitors of amino acid transporters [117] could reduce the burden of the NTBC-induced hypertyrosinemia.

## 3. A Digital Platform for AKU: ApreciseKUre

Intrinsic difficulties for assessing disease severity and response to treatment (other than HGA measurement) characterize AKU [118,119,120], while establishing a prognosis is hindered by the lack of suitable biomarkers, as the pathophysiological mechanisms of AKU still need further clarification. Hurdles common to other rare conditions, such as fragmented knowledge, dispersed expertise, geographically scattered patient populations, and redundant investigations, are to be faced in AKU studies.

Nowadays, the transformative potential of Artificial Intelligence (AI) is becoming increasingly clear in the biomedical and clinical field [121]. By filling gaps and identifying patterns in medical/clinical records, AI can inform prognosis and guide decisions in rare diseases [122,123]. Furthermore, AI can be used to develop risk prediction models based on the identification of early markers suggestive of disease progression or bad prognosis [124]. Overall, AI can assist physicians in various stages of disease management, enabling evaluation of treatment efficacy and impact on specific molecular characteristics.

Precision medicine (PM) approaches offer strategies for prevention, diagnosis, treatment, and monitoring of diseases, laying the basis for a comprehensive understanding of individual patients, a crucial step toward personalized healthcare. The collection of a critical amount of data on biological markers describing disease progression or response to specific treatment(s) is therefore imperative to aid patient stratification in rare diseases [125]. This implies the need to create dedicated registries collecting, integrating, and analysing data streams from different groups, which is central in developing a PM ecosystem, with scientists, clinicians, and patients sharing knowledge. A limit of such registries is that they are often created at the national or local level, to map prevalence/incidence and to gather information on rare diseases in selected areas. Additionally, most of the data used for such disease registries come from clinical data, observational studies, and voluntary sources. With PM being implemented in health systems across the EU, it would be ideal if further data could be gathered to strengthen these registries [126].

**Figure 4:**
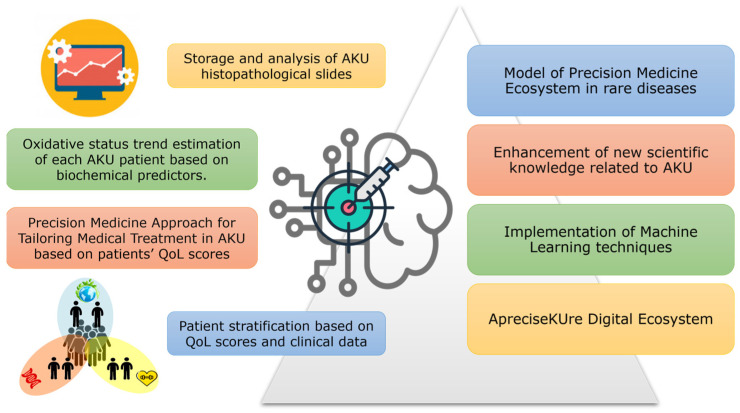
Data analysis techniques and expected outcome for ApreciseKUre.

In this context, the need of a multidisciplinary, interactive, and integrated database for AKU arose in the last years, leading to ApreciseKUre (www.bio.unisi.it/aprecisekure/, accessed on 17 June 2024; www.bio.unisi.it/aku-db/, accessed on 17 June 2024), a digital platform containing data on more than 210 AKU subjects from all over the world. Stored data include patient-derived information related to QoL, medical/research data, and *HGD* mutations [127]. With up to 110 fields per record, 82 numerical attributes, and 8 Booleans (the remaining fields being categorical values), ApreciseKUre is an easily accessible reference database for AKU. It also integrates computational predictive models able to investigate the health status of AKU patients even when mechanistic relationships between model variables cannot be determined. Figure 4 summarizes all the data analysis techniques, ranging from more common statistical data mining to deeper machine learning models, that are available so far in ApreciseKUre. Several models were implemented in ApreciseKUre with different goals, as follows [128]:

- A refreshable correlation matrix working on numerical data that might provide support for early diagnosis, monitoring, treatment, and help assessing the suitability of selected biomarkers in AKU [129]. More generally, this tool may promote a deeper understanding of AKU progression and the identification of novel prognostic biomarkers that can be used for a more efficient clinical monitoring;
- A predictive tool working on clinically measurable parameters investigating the oxidative stress status of AKU patients [130], which can help monitoring disease evolution and, possibly, lead to suitable antioxidant therapies;
- A K-nearest neighbours algorithm used to predict QoL scores starting from selected biomarkers [131].This might help in patient stratification and, in turn, addressing unresolved problems with a significant clinical impact on early diagnosis, disease prediction, and treatment outcome;
- A model correlating used drugs and scores associated to patients’ QoL [132]. This follows the idea of personalizing the treatment according to “personal” and pathological characteristics and is justified by the fact that in AKU, similarly to most rare genetic conditions, current state-of-the-art treatment is inadequate. The only drug that is currently approved for the treatment of AKU, nitisinone, can decrease urinary excretion of HGA and slow down the progression of the disease, but it comes with unwanted side effects and uncertainty on the most suitable timing to start treatment [106];
- An unsupervised clustering method able to stratify AKU population into subgroups with similar features to obtain a first genotype/phenotype stratification of AKU subjects and investigate the distribution of *HGD* mutations across the obtained clusters [133];
- A plugin named AKUImg, the first AKU-dedicated image repository, created for the storage and analysis of AKU histopathological slides [134]. By providing clinicians and researchers with a tool that can differentiate between AKU and healthy cartilage slides, it might help the scientific community to screen slides for an extremely rare condition such as AKU. The plugin is indeed integrated with an accurate predictive model based on a standard image processing approach, able to distinguish the presence of AKU by comparing histopathological images.

## 4. Conclusions and Future Perspectives

AKU manifestations are caused by the inefficiency of the enzyme HGD to catabolize HGA and its consequential life-long accumulation (if untreated) in the body. Excess HGA leads to ochronosis, a phenomenon involving aggregation, oxidation, and inflammation, with serious detrimental effects on affected tissues and organs. The early onset of OA-like symptoms due to ochronosis seriously reduces patients’ QoL.

In the last two decades, several cell- and tissue-based AKU models offered novel molecular insights into AKU. These findings could be instrumental for the development of novel therapies to be administered along with NTBC, which is currently the only approved drug to treat AKU but still an imperfect treatment. Also, such novel therapies may be considered for early stages of the disease, representing additional resources to counteract oxidative stress, inflammation, amyloidosis as well as HGA-induced molecular alterations of cellular components.

Strategies and networks pooling knowledge about rare diseases are amenable to information technology, particularly AI and machine learning [135]. However, further challenges are expected for rare diseases, including key organizational issues for data collection and storage, such as evaluation of the quality of data, harmonization and standardization strategies, creation of best practices to guarantee data protection, ownership, and control [135]. Due to the costs related to data collection and processing tasks, and to allow the data to be interoperable, discoverable, and accessible, sustainability issues are expected as well [135,136]. Regulatory restrictions will also be needed for the use of AI tools on such datasets [135]. Additional problems can be expected specifically for AKU, including suitable patient recruitment strategies (AKU is usually a non-fatal disease), incomplete data (due to limited access to specialized tests in different geographical areas), collection of longitudinal data (being that AKU is a life-long disease, continuous patient engagement is needed), and lack of data in the pre-symptomatic stage (disabling ochronotic arthropathy appears around the fourth decade of life).

Strategies and networks pooling knowledge about rare diseases are amenable to information technology, particularly AI and machine learning [135]. However, further challenges are expected for rare diseases, including key organizational issues for data collection and storage, such as evaluation of the quality of data, harmonization and standardization strategies, creation of best practices to guarantee data protection, ownership, and control [135]. Due to the costs related to data collection and processing tasks, and to allow the data to be interoperable, discoverable, and accessible, sustainability issues are expected as well [135,136]. Regulatory restrictions will also be needed for the use of AI tools on such datasets [135]. Additional problems can be expected specifically for AKU, including suitable patient recruitment strategies (AKU is usually a non-fatal disease), incomplete data (due to limited access to specialized tests in different geographical areas), collection of longitudinal data (being that AKU is a life-long disease, continuous patient engagement is needed), and lack of data in the pre-symptomatic stage (disabling ochronotic arthropathy appears around the fourth decade of life).

In this review, we have shown how the use of computational methods and dedicated digital resources incorporating AI tools might provide a chance to transform AKU-related data (obtained by both clinical and research sources) into actionable information for a PM approach. Overall, this scenario may serve as a paradigm for other (rare) conditions.

## Abbreviations

4-HPPD, 4-hydroxyphenylpyruvate dioxygenase; AKU, alkaptonuria; AOPP, advanced oxidation protein products; AI, artificial intelligence; ECM, extracellular matrix; HGA, homogentisic acid; HGD, homogentisate 1,2-dioxygenase; Hh, Hedgehog; NTBC, nitisinone; OA, osteoarthritis; PM, precision medicine; PTI, protein thiolation index; PTMs, post-translational modifications; QoL, quality of life; RA, rheumatoid arthritis; ROS, reactive oxygen species; SAA, serum amyloid A; SAP, serum amyloid P; Smo, Smoothened.
